# Geographic patterns of *Lucanus* (Coleoptera: Lucanidae) species diversity and environmental determinants in China

**DOI:** 10.1002/ece3.6911

**Published:** 2020-10-20

**Authors:** Dan Chen, Li‐Jun Cao, Jin‐Ling Zhao, Xia Wan, Shu‐Jun Wei

**Affiliations:** ^1^ School of Resources and Environmental Engineering Anhui Province Key Laboratory of Wetland Ecosystem Protection and Restoration Anhui University Hefei China; ^2^ Institute of Plant and Environmental Protection Beijing Academy of Agriculture and Forestry Sciences Beijing China; ^3^ National Engineering Research Center for Agro‐Ecological Big Data Analysis & Application Anhui University Hefei China

## Abstract

Clarifying the geographic patterns of species diversity and the determinant factors can provide essential information for species conservation and management. Stag beetles (Coleoptera: Lucanidae) of *Lucanus* are important saproxylic insects and can be used for biomonitoring forests. Most of *Lucanus* species are facing conservation concerns due to their limited distribution and fragmented habitats, particularly in China, which has the richest species diversity of this genus. The distribution patterns of species diversity of *Lucanus* at large spatial scales remain portly understood. We studied the distribution patterns of *Lucanus* and its environmental and geographic determinants in China. Distribution data for 72 species and subspecies were examined. All these species are distributed in southern China except for *Lucanus maculifemoratus dybowskyi*, which is mainly distributed in north China. The hotspot for *Lucanus* in China is southeastern Tibet. Our study indicated that the species richness of *Lucanus* in China was shaped by the precipitation of the wettest and driest month, net primary productivity, digital elevation model, and latitude at a large scale. These variables collectively explained 56.2% of the variation in species richness; precipitation contributed the most (44.1%). Our results provide valuable insights to improve the conservation of *Lucanus* and can contribute to furthering our understanding of the biogeography of stag beetles in China.

## Graphical abstract

We describe the diversity of the stag beetle species and further explore its formation. The result indicated that the variables of geography vegetation, temperature, and precipitation collectively shaped the distributed pattern, and geographical variables are most important.

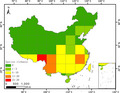

## 1 INTRODUCTION

Understanding the large‐scale geographic patterns of species diversity and the forces driving these patterns is a central topic in ecology and biogeography (Gaston, 2000; James & Mark, 2000; Ornelas et al., 2015; Santos et al., 2011). Investigating such patterns, can provide important information for regional species protection and management (Chesson, 2000; Gaston, 2000; Leibold & McPeek, 2006; Stanley et al., 2014).The distribution pattern of species richness has been investigated at large spatial scale (Brown, 1984; Gaston, 2000; Santos et al., 2011) for plants (Freestone & Inouye, 2006; Matthews et al., 2019; Qian & Song, 2013), birds (Hawkins et al., 2006; Kissling et al., 2012), mammals (Chen et al., 2017; Lin et al., 2015; Marcelo & Douglas, 2004), insects (Shen et al., 2016; Zhang et al., 2018), and bacteria (Fuhrman et al., 2008). Latitude was the most common and significant factor influencing species diversity (Gaston, 2000; Rosenzweig, 1992). However, exceptions were found, for example, species distribution patterns varied with area and in different groups (Noah & Jackson, 2006; Silva & Brandao, 2014; Wang et al., 2019).

Hypotheses on the factors shaping large‐scale species diversity have been proposed (Brown et al., 2004; Hubbell, 2001; Palmer, 1994). The environmental heterogeneity hypothesis based on climatic factors suggests that higher environmental heterogeneity promotes a number of habitat types and enhances species diversity (David, 1991; Lin et al., 2013; Stein et al., 2014; Tews et al., 2004). Increasingly, studies have found that geographic distance was the primary factor driving species diversity (Freestone & Inouye, 2006; Legendre et al., 2005; Leigh et al., 2004; Murphy et al., 2016). However, studies on leaf beetles (Coleoptera: Chrysomelidae) indicated that the effects of environment and geographic position were similar (Baselga & Jiménez‐Valverde, 2007).

Stag beetles (Coleoptera: Lucanidae) are saproxylic insects that commonly inhabit the lowlands or mountains dominated by broadleaf woody plants. They play important roles in the carbon cycle because their larvae live in and feed on decaying wood, and adults feed on tree sap or decaying fruit (Songvorawit et al., 2017; Tanahashi et al., 2009). Lucanidae species have been used as forest biodiversity indicators in Europe because temperature and deadwood significantly affect the presence of European stag beetles (Lachat et al., 2012; Thomaes et al., 2008). Climate has been speculated as the main driving factor for *Colophon* (Lucanidae) evolution (Switala et al., 2014). Morphological evidence has suggested that the evolution of mandibles of stag beetles was related to environmental heterogeneity rather than genetic differentiation (Huang & Lin, 2010; Shingleton et al., 2011). These studies on a limited number of species showed that environmental factors are critical to stag beetles. Nevertheless, the diversity distribution patterns of stag beetles at large scales remain poorly understood. More works are necessary to explore the factors on driving distribution patterns of stag beetles. *Lucanus* is widely accepted as the most typical representative of Lucanidae. Studies of molecular data indicated that *Lucanus* diverged circa 51.3 mya (95% HPD: 48.7–53.9 mya), and its sister group is *Prismognathus* (Hosoya & Araya, 2005; Kim & Farrell, 2015). Species (including subspecies) of *Lucanus* are particularly abundant in the Oriental region, including south and southwest China, India, Laos, Vietnam, and Myanmar (Fujita, 2010; Huang & Chen, 2010; Lin, 2017; Wan, 2007). In the present study, we examined the distribution and diversity patterns of *Lucanus* species in China and quantified the relative contribution of environmental conditions and spatial factors. The results will help us to understand the species diversity and distribution of these stag beetles.

## 2 MATERIALS AND METHODS

### 2.1 Collection of species distribution data

Distribution data of *Lucanus* species in China were retrieved from three sources: volumes I–III of “Stag Beetles of China” (Huang & Chen, 2010, 2013, 2017); collecting records from 1982 to 2018 of *Lucanus* preserved in the ecological geology specimen room at Anhui University (collected insects were not listed as “protected”); collecting records from other museums and universities (Wan, 2007), for example, Zoological Museum "La Specola," University of Florence (Firenze, Italy), Museum national d’ histoire naturelle (Paris, France), Museo Civico di Storia Naturale (Milan, Italy), Natural History Museum (London, UK), Museum für Naturkunde (Berlin, Germany), Staatliches Museum für Tierkunde (Dresden, Germany), Senckenberg Deutsches Entomologisches Institut (Müncheberg), Entomological Museum of Hebei University (Baoding, China), Institute of Entomology, Chinese Academy of Sciences (Shanghai, China), Museum of Insect, Chinese Agricultural University (Beijing, China), and the Department of Biology, Shanghai Normal University (Shanghai, China). Distribution data were also obtained through literature surveys, the Global Biodiversity Information Facility (GBIF) (GBIF.org 14 January 2020) and Bio‐Nica website (http://www.bio‐nica.info/home/index.html). After removing problematic and duplicate information, we obtained 1856 distribution records for 72 species of *Lucanus*. Although we attempted to obtain available records for different species, the sampling effort was inevitably uneven.

### 2.2 Environmental and geographic data

To analyze the association between species diversity and environmental and geographic factors, we studied 24 variables (Table S1): 19 bioclimatic variables including temperature and precipitation; two vegetation variables: normalized difference vegetation index (NDVI) and net primary productivity (NPP); and three geographic factors: digital elevation model (DEM), latitude (LAT), and longitude (LON). Data for 19 climate variables were obtained from WorldClim version 2 with a spatial resolution of 5 min (Fick & Hijmans, 2017). NDVI (Xu, 2018), NPP, and DEM data were obtained from the Resource and Environment Data Cloud Platform (http://www.resdc.cn). LAT and LON were transformed from species distribution information.

We extracted raster data in ArcGIS 10.2, including reprojection using Lambert conformal conic projection, resampling, extracting the value by distribution point, and calculating the value in each cell (500 × 500 km^2^). Among them, 19 bioclimatic variables were characterized by the range (maximum minus minimum) of values of all distribution points in each cell. Similarly, NPP, NDVI, LAT, and LON were characterized by the mean of values; DEM was characterized by the standard deviation of values.

### 2.3 Statistical analysis

Species accumulation curves (SAC) were widely used to assess the completeness of sampling effort or sampling completeness (Colwell et al., 2004; Mongombe et al., 2019). To estimate the sampling completeness of *Lucanus* species in China at 500 × 500 km^2^, we constructed SACs based on the number of samples (each grid represented a sample). The function “specaccum” in the package *vegan* (Oksanen et al., 2013) in R 3.6.1 (R‐Core‐Team, 2019) was used to find the SAC with the classic method "random," which adds grids in a random order. The number of permutations was set to 100. To eliminate the effect of the square of the study area on species richness, we used an equal‐area grid cell size of 500 × 500 km^2^ (Ding et al., 2006; Marcelo & Douglas, 2004). We overlapped all species distributions with the 500 × 500 km^2^ fishnet and then counted the number of species present within each grid as the species richness values for each grid. Drawing fishnets and calculating species richness were carried out in ArcGIS 10.2.

We filtered the 24 variables through Pearson's correlation analysis (|*r*| > 0.8) to eliminate the strong correlation between environmentally heterogeneous variables (Garg & Tai, 2012). Pearson's correlation analysis was performed in IBM SPSS Statistics v24. To assess the relationship between species richness and environmental variables, we applied generalized linear modeling (GLM) with Poisson regression and negative binomial regression as those models are typically used for count data. We validated the models by applying the log‐likelihoods test and the null hypothesis to compare the Poisson and negative binomial GLMs. Regression models and model validation were carried out in R; Poisson GLMs were constructed by the function “glm” in the *vegan* package; negative binomial GLMs were constructed by the function “glm.nb” in the *MASS* package (Venables & Ripley, 2002); model selection process was by the function “drop1,” all variables being significant at the 5% level suggested that the model selection was finished; model validation was conducted by the function “odTest” in the *pscl* package (Zeileis et al., 2008).

## 3 RESULTS

### 3.1 Distribution of species diversity

**Figure 1:**
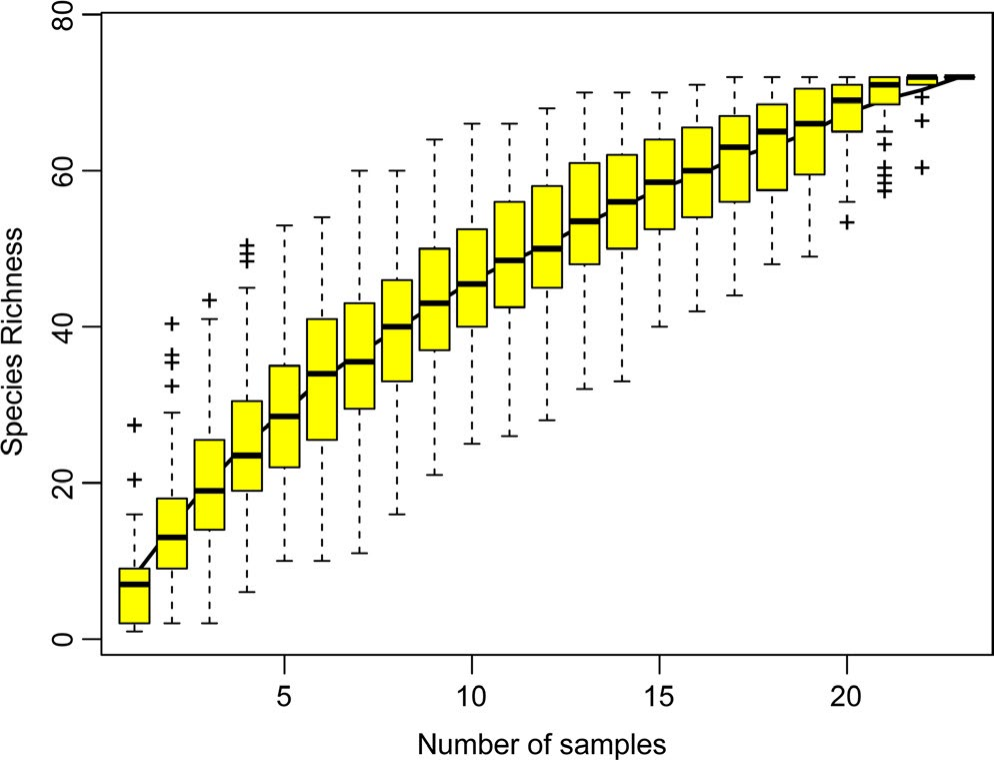
Sample‐based species accumulation curves of *Lucanus* species in China

The SAC illustrates trends in species richness with changing number of samples. As the number increases, the SAC increases sharply at first and then slowly, indicating sufficient sampling for data analysis (Figure 1).

**Figure 2:**
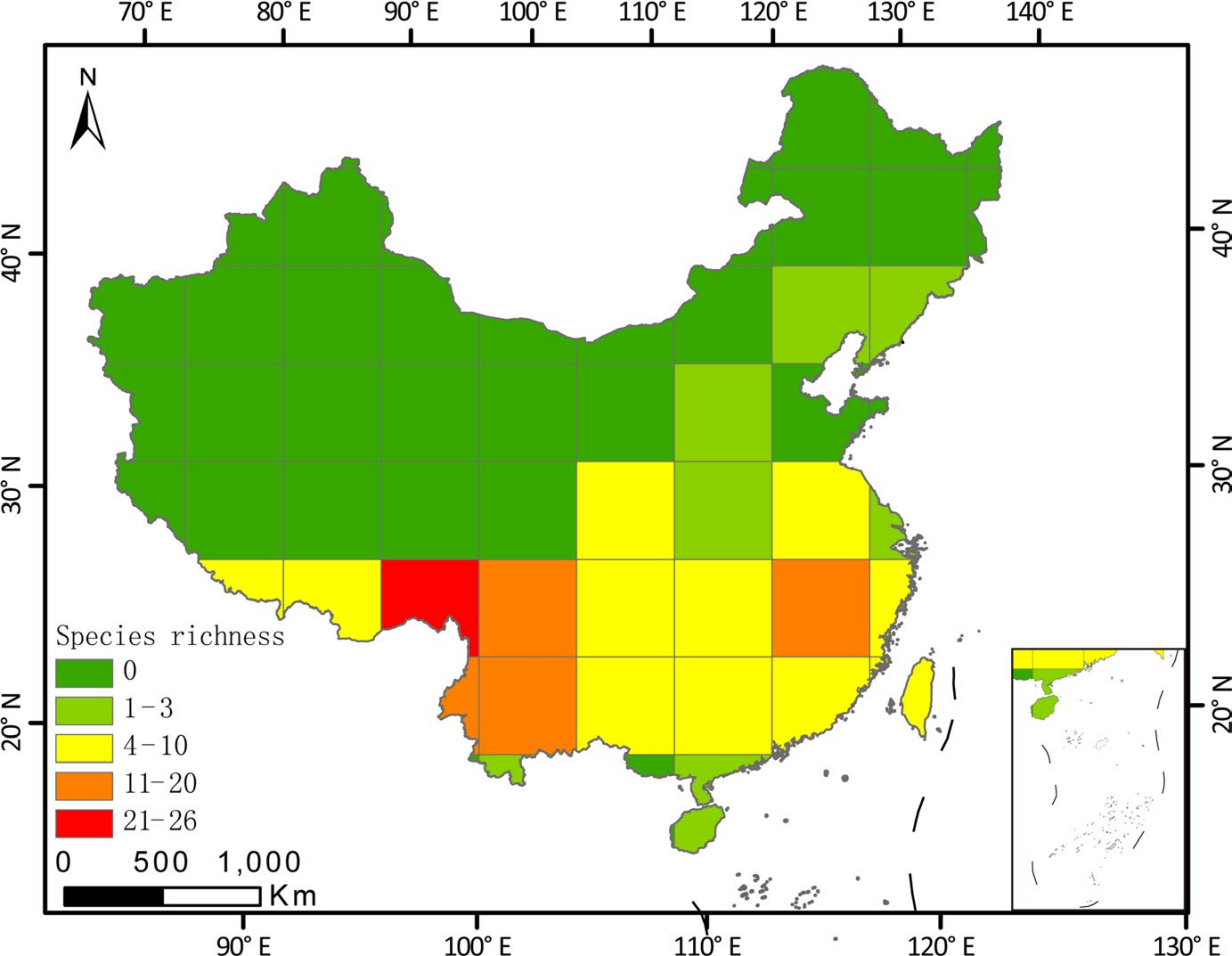
Species richness of *Lucanus* in 500 × 500 km^2^ grid cells in China. Different colors indicate the range of species richness

In China, the distribution range of *Lucanus* is 18–43°N and 85–127°E. Seventy‐two species of stag beetle species were found (Figure 2). Species richness of the *Lucanus* species differed significantly between the south (Oriental) and north (Palaearctic) of China. All *Lucanus* species were distributed in the Oriental realm of China except for *Lucanus maculifemoratus dybowskyi*. This species is distributed across the south and north, but is mainly distributed in the north and northeast of China. Based on grids of 500 × 500 km^2^, we revealed southeastern Tibet as a hotspot area where the highest species richness of *Lucanus* was observed (26 species). Fewer grids had high species richness, and many species were only distributed in one or two grids, presenting a narrower distribution. For instance, eight species (*L. datunensis, L. formosanus, L. kanoi, L. kurosawai, L. m. taiwanus, L. miwai, L. ogakii*, and *L. swinhoei*) were endemic to Taiwan, and *L. datunensis* only occurred at Datunshan within the Yangmingshan National Park; *L. fanjingshanus* only occurred at Fanjingshan; eight species were endemic to Yunnan; and 18 species were endemic to south Tibet (mainly distributed in southeast Tibet).

### 3.2 Environmental and spatial associations

Nine variables selected through the correlation analysis, namely, annual mean temperature, temperature seasonality, precipitation of wettest month (PWM), precipitation of driest month (PDM), NDVI, NPP, LAT, LON, and DEM (Table S2). Further model selection of Poisson GLMs only retained five variables (Table 1). All of these terms in the model were significant (*p* < .01). The output of the negative binomial GLM was similar to the Poisson GLM output (Table 2), except that the corresponding significance level was lower, and the AIC was slightly higher. The chi‐square test statistic was equal to 1.5972, and the *p*‐value was equal to .1031 (*p* > .05), which did not support the null hypothesis. Hence, there was strong support for the Poisson GLM.

**Table 1:** Regression analysis results for environmental variables regressed against species richness with Poisson general linear models

	Estimate	*SE*	*z* value	*P* (>|*z*|)	Signif. codes
(Intercept)	2.589568	0.665381	3.892	9.95E−05	***
PWM	0.008224	0.001121	7.339	2.16E−13	***
PDM	−0.01479	0.002864	−5.164	2.42E−07	***
NPP	0.000137	4.13E−05	3.31	0.000935	***
DEM	0.00119	0.000359	3.317	0.000909	***
LAT	−0.06731	0.025237	−2.667	0.007649	**

Note

Signif. codes: “***” indicated that 0 < *p* < .001; “**” indicated that 0.001 < *p* < .01; Null deviance: 122.152 on 22 degrees of freedom; Residual deviance: 33.894 on 17 degrees of freedom; AIC: 125.78.

**Table 2:** Regression analysis results for environmental variables regressed against species richness with negative binomial general linear models

	Estimate	*SE*	*z* value	Pr(>|*z*|)	Signif. codes	
(Intercept)	2.55E+00	7.89E−01	3.234	0.00122		**
PWM	8.03E−03	1.48E−03	5.415	6.12E−08		***
PDM	−1.46E−02	3.58E−03	−4.062	4.86E−05		***
NPP	1.23E−04	5.49E−05	2.249	0.02449		*
DEM	1.28E−03	4.52E−04	2.825	0.00473		**
LAT	−6.68E−02	2.90E−02	−2.302	0.02131		*

Note

Signif. codes: “***” indicated that 0 < *p* < .001; “**” indicated that 0.001 < *p* < .01; “*” indicated that 0.01 < *p* < .05; Null deviance: 82.949 on 22 degrees of freedom; Residual deviance: 23.774 on 17 degrees of freedom; AIC: 126.18.

Abbreviations: DEM, digital elevation model; LAT, latitude; NPP, net primary productivity; PDM, precipitation of driest month; PWM, precipitation of wettest month.

**Figure 3:**
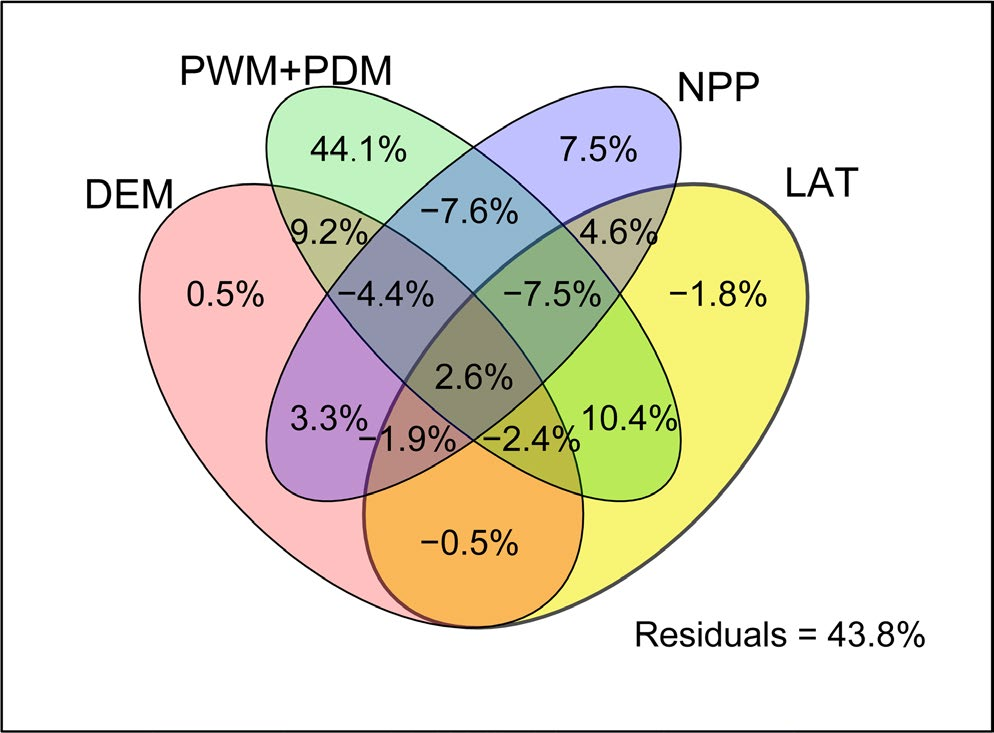
Results of variation partitioning for *Lucanus* species richness. Variation partitioning according to four matrixes. PWM, precipitation of wettest month; PDM, precipitation of driest month; NPP, net primary productivity; DEM, digital elevation model; LAT, latitude

To further quantify the contribution of different variables to species richness, we classified these five variables into four matrixes: precipitation (PWM, PDM), NPP, LAT, and DEM for conducting variation partitioning. The variation partitioning results showed that the total contribution of these five variables was 56.2%; precipitation (PWM, PDM) independently explained 44.1% (Figure 3), which was much higher than that of several other variables; NPP independently explained 7.5%; DEM independently explained 0.5%; LAT independently explained less than zero (−0.18%).

## 4 DISCUSSION

China is the main distribution center of *Lucanus* species, but many species of this genus present a narrow distribution, which may be due to a weak dispersion ability or specific habitat needs, or even the different ecological effects caused by environmentally heterogeneous habitats (Tews et al., 2004; Thomaes, 2009; Tini et al., 2017). For example, the species distributed in Taiwan are endemic. Only *L. m. dybowskyi* occurs in north China. This is related to the strong diffusion ability of this species and its unique adaptability to the cold environment and limited broad‐leaved forests (Fang & Yoda, 1991; Qiao et al., 2015).

At large spatial scales, previous studies tended to report species richness showing a certain pattern along the latitude gradient (Archibald et al., 2016; Dunn et al., 2009; Stegen et al., 2012). In our study, due to the limited number of species and a large proportion of endemic species, we only considered the species distribution patterns at a large scale (500 × 500 km^2^). Although there was no obvious pattern along latitude, this factor still has an important effect on species richness according to the results of the regression model. However, the results of variation partitioning indicated negative explained variances. This situation could arise when certain relationships are present in the data, and no solution is available to meaningfully remove them (Peres‐Neto et al., 2006).

The other four factors included in the regression model all had positive independent contributions to the variation in species richness. Among them, precipitation contributed the most. Precipitation could have indirect effects that influence the species richness of *Lucanus* because larvae of these stag beetles live in and feed on dead wood (Songvorawit et al., 2017). Wood decay is correlated with moisture content (Crockatt & Bebber, 2015; Herrmann & Bauhus, 2013). Therefore, moderate water content enhances the occurrence and abundance of larval stag beetles (Songvorawit et al., 2017). Huang (2014) reported that climate change may threaten the survival of *Lucanus* species due to their specific habitat preferences. However, our model did not include temperature, which is unexpected.

*Lucanus* inhabit mature deciduous forests, especially oak woodland, and feed on dead wood (Bardiani et al., 2017). NPP was also one of the major factors in our model, which is an important manifestation of vegetation productivity. The species richness of *Lucanus* can be directly affected by vegetation. NPP was positively correlated with *Lucanus* species richness, which means that high vegetation productivity can provide more resources, indicating that more species can be accommodated. Furthermore, studies revealed that plant cover has a significant effect on the diversity of many other groups, such as ladybugs (Coleoptera: Coccinellidae) (Sushko, 2018), stink bugs (Heteroptera: Pentatomidae) (Reisig et al., 2015), and small mammals (Keller & Schradin, 2008). However, plant diversity is under increasing threat due to human activities (Bardiani et al., 2017; Sharrock et al., 2011). Therefore, we suggest that strategies for conserving *Lucanus* should focus on protecting habitats. An interesting example included the use of artificial habitats comprised of rotten woodpiles and buckets with rotten deciduous leaves for larvae provides us with references (New, 2005). Besides, studies on the distribution patterns of ants (Hymenoptera: Formicidae) and ground beetles (Coleoptera: Carabidae) in China reported that changes in altitude could be a key factor affecting the distribution of species diversity (Shen et al., 2016; Yang et al., 2017). This result is consistent with our results, but the independent contribution of DEM was low.

With global climate change and large‐scale deforestation, many species of *Lucanus* are facing problems from habitat fragmentation and loss. These species must be urgently protected. *L. datunensis* is the only species that is currently protected in China, and there are no protection measures or threat level assessments for the others. The distribution information for *Lucanus* in China is lacking. Therefore, our research can attract the attention of relevant researchers to undertake more effective monitoring and protection plans. Our analysis only found part of the relevant factors, and more factors (e.g., percentage of broad‐leaved forest) and smaller scales should be explored in the future.

## CONFLICT OF INTEREST

There have no competing interests exist.

## AUTHOR CONTRIBUTIONS

Dan Chen: Data curation (lead); Formal analysis (equal); Investigation (lead); Methodology (equal); Visualization (equal); Writing‐original draft (lead); Writing‐review & editing (lead). Xia Wan: Conceptualization (lead); Resources (lead); Supervision (equal); Visualization (equal); Writing‐review & editing (equal). Li‐Jun Cao and Jin‐Ling Zhao: Formal analysis (supporting); Methodology (supporting). Shu‐Jun Wei: Methodology (supporting); Writing–review and editing (supporting).

## Supporting information

Table S1

Table S2

## Data Availability

Dryad, Dataset, https://doi.org/10.5061/dryad.08kprr4zx.
