# Supplementary material for: Geographic patterns of Lucanus (Coleoptera: Lucanidae) species diversity and environmental determinants in China
Source: Ecol Evol. 2020 Oct 20;10(23):13190–7. doi: 10.1002/ece3.6911 (PMC7713949; doi:10.1002/ece3.6911)
Supplement: Supplementary file 1 — Table S1 [file ECE3-10-13190-s001.docx]

| Type | Factor | Abbreviation |
| --- | --- | --- |
| Temperature | Annual Mean Temperature | AMT |
|  | Mean Diurnal Range (Mean of monthly (max temp - min temp)) | MDR |
|  | Isothermality (BIO2/BIO7) (* 100) | ISOT |
|  | Temperature Seasonality (standard deviation *100) | TSN |
|  | Max Temperature of Warmest Month | MTWM |
|  | Min Temperature of Coldest Month | MTCM |
|  | Temperature Annual Range (BIO5-BIO6) | TAR |
|  | Mean Temperature of Wettest Quarter | MTWQ |
|  | Mean Temperature of Driest Quarter | MTDQ |
|  | Mean Temperature of Warmest Quarter | MTMQ |
|  | Mean Temperature of Coldest Quarter | MTCQ |
| Precipitation | Annual Precipitation | AMP |
|  | Precipitation of Wettest Month | PWM |
|  | Precipitation of Driest Month | PDM |
|  | Precipitation Seasonality (Coefficient of Variation) | PSN |
|  | Precipitation of Wettest Quarter | PWQ |
|  | Precipitation of Driest Quarter | PDQ |
|  | Precipitation of Warmest Quarter | PMQ |
|  | Precipitation of Coldest Quarter | PCQ |
| Vegetable | Net Primary Productivity | NPP |
|  | Normalized Difference Vegetation Index | NDVI |
| Spatial | Digital Elevation Model | DEM |
|  | longitude | LON |
|  | latitude | LAT |
