# Supplementary material for: Geographic patterns of Lucanus (Coleoptera: Lucanidae) species diversity and environmental determinants in China
Source: Ecol Evol. 2020 Oct 20;10(23):13190–7. doi: 10.1002/ece3.6911 (PMC7713949; doi:10.1002/ece3.6911)
Supplement: Supplementary file 2 — Table S2 [file ECE3-10-13190-s002.docx]

|  | | Richness | | AMT | | MDR | | ISOT | | TSN | | MTWM | | MTCM | | TAR | | MTWQ | | MTDQ | | MTMQ | | MTCQ | | AMP |
| --- | --- | --- | --- | --- | --- | --- | --- | --- | --- | --- | --- | --- | --- | --- | --- | --- | --- | --- | --- | --- | --- | --- | --- | --- | --- | --- |
| Richness | | 1 | | .594** | | .548** | | .418* | | 0.307 | | .608** | | .548** | | .428* | | .604** | | .588** | | .589** | | .562** | | .642** |
| AMT | | .594** | | 1 | | .854** | | .815** | | .729** | | .942** | | .950** | | .728** | | .961** | | .968** | | .976** | | .980** | | .835** |
| MDR | | .548** | | .854** | | 1 | | .754** | | .706** | | .795** | | .850** | | .790** | | .873** | | .835** | | .830** | | .867** | | .699** |
| ISOT | | .418* | | .815** | | .754** | | 1 | | .552** | | .742** | | .732** | | .435* | | .841** | | .777** | | .814** | | .793** | | .795** |
| TSN | | 0.307 | | .729** | | .706** | | .552** | | 1 | | .785** | | .700** | | .889** | | .797** | | .770** | | .762** | | .731** | | .449* |
| MTWM | | .608** | | .942** | | .795** | | .742** | | .785** | | 1 | | .828** | | .715** | | .953** | | .898** | | .978** | | .883** | | .749** |
| MTCM | | .548** | | .950** | | .850** | | .732** | | .700** | | .828** | | 1 | | .786** | | .886** | | .965** | | .877** | | .986** | | .807** |
| TAR | | .428* | | .728** | | .790** | | .435* | | .889** | | .715** | | .786** | | 1 | | .765** | | .785** | | .704** | | .771** | | .526** |
| MTWQ | | .604** | | .961** | | .873** | | .841** | | .797** | | .953** | | .886** | | .765** | | 1 | | .921** | | .968** | | .930** | | .807** |
| MTDQ | | .588** | | .968** | | .835** | | .777** | | .770** | | .898** | | .965** | | .785** | | .921** | | 1 | | .924** | | .982** | | .828** |
| MTMQ | | .589** | | .976** | | .830** | | .814** | | .762** | | .978** | | .877** | | .704** | | .968** | | .924** | | 1 | | .925** | | .783** |
| MTCQ | | .562** | | .980** | | .867** | | .793** | | .731** | | .883** | | .986** | | .771** | | .930** | | .982** | | .925** | | 1 | | .826** |
| AMP | | .642** | | .835** | | .699** | | .795** | | .449* | | .749** | | .807** | | .526** | | .807** | | .828** | | .783** | | .826** | | 1 |
| PWM | | .635** | | .790** | | .640** | | .730** | | 0.351 | | .651** | | .795** | | .485* | | .739** | | .773** | | .715** | | .804** | | .935** |
| PDM | | 0.073 | | .531** | | .515* | | .748** | | 0.263 | | .449* | | .494* | | 0.191 | | .506* | | .517* | | .494* | | .528** | | .657** |
| PSN | | .441* | | .817** | | .698** | | .845** | | 0.387 | | .691** | | .759** | | 0.393 | | .750** | | .776** | | .750** | | .813** | | .872** |
| PWQ | | .702** | | .766** | | .601** | | .664** | | 0.354 | | .652** | | .767** | | .496* | | .721** | | .760** | | .697** | | .770** | | .931** |
| PDQ | | 0.104 | | .538** | | .523* | | .747** | | 0.275 | | .464* | | .500* | | 0.202 | | .518* | | .526** | | .501* | | .532** | | .662** |
| PMQ | | .621** | | .756** | | .632** | | .696** | | 0.323 | | .625** | | .766** | | .470* | | .708** | | .738** | | .675** | | .763** | | .935** |
| PCQ | | 0.177 | | .583** | | .540** | | .783** | | 0.324 | | .520* | | .535** | | 0.228 | | .565** | | .580** | | .552** | | .569** | | .707** |
| NDVI | | 0.277 | | 0.359 | | 0.348 | | 0.325 | | 0.401 | | 0.407 | | 0.298 | | 0.377 | | 0.397 | | 0.341 | | 0.387 | | 0.327 | | .417* |
| NPP | | -0.113 | | -0.235 | | -0.01 | | -0.153 | | -0.03 | | -0.058 | | -0.32 | | -0.063 | | -0.126 | | -0.238 | | -0.154 | | -0.295 | | -0.227 |
| DEM | | 0.328 | | .800** | | .773** | | .610** | | .722** | | .768** | | .725** | | .652** | | .784** | | .755** | | .794** | | .762** | | .469* |
| LON | | -0.385 | | -0.179 | | 0.059 | | 0.072 | | 0.264 | | -0.058 | | -0.186 | | 0.066 | | -0.047 | | -0.118 | | -0.129 | | -0.144 | | -0.2 |
| LAT | | -0.282 | | -0.043 | | -0.018 | | -0.215 | | 0.211 | | -0.034 | | 0.005 | | 0.136 | | -0.093 | | -0.011 | | -0.041 | | 0.003 | | -0.283 |
|  | PWM | | PDM | | PSN | | PWQ | | PDQ | | PMQ | | PCQ | | NDVI | | NPP | | DEM | | LON | | LAT | |  |  |
| Richness | .635** | | 0.073 | | .441* | | .702** | | 0.104 | | .621** | | 0.177 | | 0.277 | | -0.113 | | 0.328 | | -0.385 | | -0.282 | |  |  |
| AMT | .790** | | .531** | | .817** | | .766** | | .538** | | .756** | | .583** | | 0.359 | | -0.235 | | .800** | | -0.179 | | -0.043 | |  |  |
| MDR | .640** | | .515* | | .698** | | .601** | | .523* | | .632** | | .540** | | 0.348 | | -0.01 | | .773** | | 0.059 | | -0.018 | |  |  |
| ISOT | .730** | | .748** | | .845** | | .664** | | .747** | | .696** | | .783** | | 0.325 | | -0.153 | | .610** | | 0.072 | | -0.215 | |  |  |
| TSN | 0.351 | | 0.263 | | 0.387 | | 0.354 | | 0.275 | | 0.323 | | 0.324 | | 0.401 | | -0.03 | | .722** | | 0.264 | | 0.211 | |  |  |
| MTWM | .651** | | .449* | | .691** | | .652** | | .464* | | .625** | | .520* | | 0.407 | | -0.058 | | .768** | | -0.058 | | -0.034 | |  |  |
| MTCM | .795** | | .494* | | .759** | | .767** | | .500* | | .766** | | .535** | | 0.298 | | -0.32 | | .725** | | -0.186 | | 0.005 | |  |  |
| TAR | .485* | | 0.191 | | 0.393 | | .496* | | 0.202 | | .470* | | 0.228 | | 0.377 | | -0.063 | | .652** | | 0.066 | | 0.136 | |  |  |
| MTWQ | .739** | | .506* | | .750** | | .721** | | .518* | | .708** | | .565** | | 0.397 | | -0.126 | | .784** | | -0.047 | | -0.093 | |  |  |
| MTDQ | .773** | | .517* | | .776** | | .760** | | .526** | | .738** | | .580** | | 0.341 | | -0.238 | | .755** | | -0.118 | | -0.011 | |  |  |
| MTMQ | .715** | | .494* | | .750** | | .697** | | .501* | | .675** | | .552** | | 0.387 | | -0.154 | | .794** | | -0.129 | | -0.041 | |  |  |
| MTCQ | .804** | | .528** | | .813** | | .770** | | .532** | | .763** | | .569** | | 0.327 | | -0.295 | | .762** | | -0.144 | | 0.003 | |  |  |
| AMP | .935** | | .657** | | .872** | | .931** | | .662** | | .935** | | .707** | | .417* | | -0.227 | | .469* | | -0.2 | | -0.283 | |  |  |
| PWM | 1 | | .522* | | .854** | | .981** | | .526* | | .972** | | .556** | | 0.306 | | -.474* | | 0.367 | | -0.333 | | -0.233 | |  |  |
| PDM | .522* | | 1 | | .773** | | .418* | | .997** | | .533** | | .980** | | 0.215 | | 0.069 | | .415* | | 0.271 | | -0.208 | |  |  |
| PSN | .854** | | .773** | | 1 | | .783** | | .774** | | .830** | | .777** | | 0.315 | | -0.268 | | .558** | | -0.099 | | -0.147 | |  |  |
| PWQ | .981** | | .418* | | .783** | | 1 | | .427* | | .975** | | .479* | | 0.317 | | -.443* | | 0.341 | | -0.388 | | -0.252 | |  |  |
| PDQ | .526* | | .997** | | .774** | | .427* | | 1 | | .544** | | .986** | | 0.22 | | 0.068 | | .426* | | 0.274 | | -0.224 | |  |  |
| PMQ | .972** | | .533** | | .830** | | .975** | | .544** | | 1 | | .585** | | 0.308 | | -0.39 | | 0.363 | | -0.33 | | -0.3 | |  |  |
| PCQ | .556** | | .980** | | .777** | | .479* | | .986** | | .585** | | 1 | | 0.237 | | 0.057 | | .463* | | 0.246 | | -0.246 | |  |  |
| NDVI | 0.306 | | 0.215 | | 0.315 | | 0.317 | | 0.22 | | 0.308 | | 0.237 | | 1 | | 0.077 | | 0.27 | | -0.091 | | -0.255 | |  |  |
| NPP | -.474* | | 0.069 | | -0.268 | | -.443* | | 0.068 | | -0.39 | | 0.057 | | 0.077 | | 1 | | 0.028 | | 0.305 | | -0.225 | |  |  |
| DEM | 0.367 | | .415* | | .558** | | 0.341 | | .426* | | 0.363 | | .463* | | 0.27 | | 0.028 | | 1 | | -0.023 | | 0.075 | |  |  |
| LON | -0.333 | | 0.271 | | -0.099 | | -0.388 | | 0.274 | | -0.33 | | 0.246 | | -0.091 | | 0.305 | | -0.023 | | 1 | | 0.333 | |  |  |
| LAT | -0.233 | | -0.208 | | -0.147 | | -0.252 | | -0.224 | | -0.3 | | -0.246 | | -0.255 | | -0.225 | | 0.075 | | 0.333 | | 1 | |  |  |
